# Minimum InDel pattern analysis of the Zika virus

**DOI:** 10.1186/s12864-018-4935-z

**Published:** 2018-07-13

**Authors:** Hyeji Lee, Mai Phuong Nguyen, Yunhee Choi, Yong-Hak Kim

**Affiliations:** Department of Microbiology, Daegu Catholic University School of Medicine, Daegu, 42472 Republic of Korea

**Keywords:** Zika virus, Minimum InDel, Polyprotein, Envelope protein, Virus typing

## Abstract

**Background:**

The Zika virus (ZIKV) can cause microcephaly and congenital abnormalities in the foetus. Recent studies have provided insights into the evolution of ZIKV from the current and previous outbreaks, but the types have not been determined.

**Results:**

We analysed the insertions and deletions (InDels) in 212 ZIKV polyproteins and 5 Dengue virus (DENV) reference sequences. Spearman correlation tests for the minimum InDel (minInDel) patterns were used to assess the type of polyprotein. Using the minInDel frequencies calculated from polyproteins with 11 elements, likelihood estimation was conducted to correct the evolutionary distance. The minInDel-corrected tree topology clearly distinguished between the ZIKV types (I and II) with a unique minInDel character in the E protein. From the 10-year average genetic distance, the African and Asian lineages of ZIKV-II were estimated to have occurred ~ 270 years ago, which is unlikely for ZIKV-I.

**Conclusions:**

The minInDel pattern analysis showed that the minInDel in the E protein is targetable for the rapid detection and determination of the virus types.

**Electronic supplementary material:**

The online version of this article (10.1186/s12864-018-4935-z) contains supplementary material, which is available to authorized users.

## Background

Zika virus (ZIKV) is a single-stranded positive RNA virus species that belongs to the genus *Flavivirus*. It was first isolated from a sentinel monkey in the Zika forest of Uganda in 1947, and the investigations revealed a mosquito-borne arbovirus that had been transmitted to animals and humans by infected *Aedes* mosquitos [1]. Most cases of human infections by ZIKV are asymptomatic (~ 80%), but some patients have mild fever, rash, joint pain, red eyes, and headache [2]. Over the past decade, Asian ZIKV outbreaks in the Pacific islands have been reported to be associated with Guillain-Barré syndrome [3]. The spread of ZIKV in Brazil and several countries of South and Central America and the Caribbean is of great concern because it can cause microcephaly and congenital abnormalities in babies whose mothers were infected during pregnancy [4–6]. Recent genomic studies have provided insights into the evolutionary patterns of ZIKV strains from the current and previous outbreaks [4, 7–10]; however, the virus types were not determined.

ZIKV genomes differentiated into African and Asian lineages, as determined by phylogenetic studies [7, 8, 11]. The African strains include relatively old strains and recombinant strains derived from co-circulating viruses in different lineages of East and West Africa areas [8, 10–12], whereas the Asian strains similar to prototype Malaysia/1966 strain P6–740 (GenBank accession no. KX377336) are the most prevalent virus in Asian Pacific American cases, with morbidity to Guillain-Barré syndrome and brain disorder [13]. Previous phylogenetic studies showed that the Asian lineage has been gradually evolving and spreading through the Asian Pacific American outbreaks, after the divergence from an African lineage had occurred ~ 50–180 years ago [10, 12, 14]. Recent phylogenetic trees of ZIKV showed a strong evolutionary bias towards a greater number of Asian strains than African strains. The molecular patterns show that recombination is likely to occur in the envelope E protein, and the nonstructural proteins NS2B and NS5 are from different viruses that co-circulate in Africa [8, 12, 15], but finding insights is still a struggle because recombination in ZIKV is a rare event in the entire genome sequence [16]. Although there is a significant increase in the number of ZIKV genomes, we still lack an understanding of the evolutionary relationships between the types, which were produced by insertion and deletion (InDel) in the viral polyprotein sequences. InDels occur to a lesser degree than substitutions in viruses as well as in cells [17, 18]. Their occurrence represents a loss of function, independent of the evolutionary time for all of the sequences and is likely to increase an active subspace (length), where their orthologues are separated prior to the high-frequency substitutions under selective constraints. The associated analysis is one of the most difficult problems because of computing the length of a sequence or retrieving its elements in a phylogenetic analysis. Therefore, we must reduce the distribution of the composition bias between the true InDels and the alignment of finite sequences.

This study aims at analysing the minimum InDel (minInDel) patterns of closely related ZIKV and Dengue virus (DENV) on the basis of a consensus polyprotein sequence to reduce the burdens of ambiguous InDels and unreliable substitutions in aligned sequences for a divergent population. To compare the minInDel patterns, independent of the parametric information on the lengths of the sequences, we performed the Spearman correlation test, after normalising the length of each protein component with the maximum length of the consensus. We introduced the minInDel frequency to adjust a timer for inferring the evolutionary distance between the sequences by the maximum likelihood method, and we showed that the virus types differentiate from each other with a unique minInDel pattern in the envelope E protein.

## Methods

### Datasets

We collected 212 ZIKV genomes that coded for the full polyproteins from the National Center for Biotechnology Information up to May 9th, 2018 (Additional file 1). Five DENV genomes were included as reference sequences that are closely related to ZIKV. Annotations of protein families were generated using the Virus Pathogen Resource (ViPR) at www.viprbrc.org. The maximum length of the viral polyprotein was determined by the sum of the maximum lengths of the individual protein components in the datasets (Additional file 2). Using the unweighted consensus, the minInDel positions were searched by Clustal [19], and the minInDel maps were produced with Geneious version 10.2.3 [20]. The alignment is evaluated by the BLOSUM 62 algorithm to mitigate any query with low similarity (< 62% conserved amino acid residues) in a protein homology group across species. Evolutionary analyses were conducted in MEGA7 [21].

### Spearman correlation between normalised protein lengths

The minInDel maps of the collected ZIKV and DENV strains were based on a consensus polyprotein sequence of 3425 amino acids. A full polyprotein is composed of 11 concatenated protein components: anchored capsid protein ancC, membrane glycoprotein prM, envelope protein E, nonstructural protein NS1, nonstructural protein NS2A, nonstructural protein NS2B, nonstructural protein NS3, nonstructural protein NS4A, protein 2 K, nonstructural protein NS4B, and RNA polymerase NS5. The ancC and prM proteins are precursors of capsid C, protein pr, and membrane glycoprotein M. Because some amino acid residues are eliminated by the maturation processes of capsid C in both ZIKV and DENV and membrane glycoprotein M in DENV, the precursor sequences of ancC and prM were used in the minInDel pattern analysis. Because InDel produces a gapped sequence, it can violate the assumptions of the molecular clock. To resolve the phylogeny, we analysed the minInDel pattern on the basis of the unweighted protein consensus sequence, which is defined as the average by a non-parametric method using the Spearman correlation [22]. To compare the rankings presented by the minInDel pattern, the protein length was normalised in a range between 0 and 1 by dividing it by the consensus which corresponds to the maximum length. Spearman’s correlation coefficient (*ρ*) for the average ranks in a set of normalised length data was calculated with the *P*-value to assess the statistical evidence for the monotonic relationship between the consensus rank and the ranks produced by the minInDel.

### Estimation of the minInDel frequency and evolutionary distance for the polyprotein

The frequency *F*_indel_ of the minInDel length in a polyprotein with *n* components (*n* = 11 in this study) was estimated by the mean root square ratio to the amino acid changes in each protein component i, including the number of minInDels (*I*_A_) and the number of substituted (*S*_A_) amino acids.

1 $$ {F}_{indel}=\frac{1}{n}\sum \limits_{i=1}^n\sqrt{{\left(\frac{I_A}{I_A+{S}_A}\right)}_i^2} $$

This value is characteristic of discrete minInDel variables in gapped sequences. The valid value ranges between zero and one, where a value of zero indicates no minInDel amino acid in the aligned sequences, and increasing values of the minInDel frequency imply that the lengths are unequal. However, identical sequences, which result in both *I*_A_ and *S*_A_ values of zero, give no information on the evolution as well as the minInDel frequency. Although the minInDel value is not obtained from a conventional computer program for the construction of a phylogenetic tree based on a multiple sequence alignment (MSA) of all existing sequences in the given dataset, it is simple to calculate the number of pure InDel amino acids by the differences in the lengths of the sequences. The measurement of realistic gaps in a pairwise alignment (only where the minInDel length was non-zero) could minimise the frequency of independent random InDel variables on the evolutionary path through the accumulation of mutations as well as the burden of computation.

From the minInDel pattern analysis using virus type-consensus sequences, individual minInDel variables are assigned to a light chain of one sequence (*C*_*L*_) aligned with the other heavy chain (*C*_*H*_), which results in an absolute InDel length difference, *δ* = *C*_*H*_ - *C*_*L*_. Because we cannot identify deletion or insertion to generate two sequences, this step is performed to reduce the InDel ambiguity in the alignment outcomes. Given that multiple minInDel variables (*x*) have independently occurred in *k* loci of a protein component, the distribution *f(δ)* of the minInDel length is related to the probability *p*(*x*) of the protein length *L* accumulating or losing random independent minInDel variables. In this case, the sum of the independent random variables can be calculated to have a probability using the Poisson binomial distribution by the discrete convolution for finite sequences, as described previously [17].

2 $$ f\left(\delta \right)={\sum}_{j=1}^kf\left(\delta -{x}_j\right)p\left({x}_j\right) $$

Previous studies have demonstrated that the estimated mutation rates of Asian strains are higher than African strains [23, 24]. In other words, the evolutionary rate of ZIKV over time is unstable. The evolutionary history could be further complicated by genetic drift through the accumulation of deleterious mutations during the systematic virus infection of mosquito vectors, which leads to decreased fitness during host switching [25], or by epistatic interactions for adaptive molecular evolution [26]. To address the cumulative effect of mutational biases of different vectors and hosts on the minInDel distribution, we used the frequency Findel in a likelihood formula.

3 $$ f\left(\delta \right)=\sum \limits_{j=1}^kf\left(\delta -{x}_j\right)\frac{{\left(L{F}_{indel}\right)}^k}{k!}\exp \left(-L{F}_{indel}\right),{F}_{indel}>0 $$

in which L is the average length of the proteins of two types (CH + CL/2). If the minInDel pattern is an invariant of the polyprotein with n protein components, the likelihood estimate $\widehat{\ell}$(Findel) between the virus type-consensus sequences was calculated by the following Eq. (4).

4 $$ \widehat{\ell}\left({F}_{indel}\right)={\prod}_{i=1}^n\sum \limits_{j=1}^kf\left({\delta}_i-{x}_j\right)\frac{{\left({L}_i{F}_{indel}\right)}^k}{k!}\exp \left(-{L}_i{F}_{indel}\right) $$

Estimating the frequency of the minInDel length (Findel) can reduce ambiguous InDel variables in the alignment of divergent sequences for the virus types, without altering the length of the protein or polyprotein. A valid unique minInDel parameter has the ability to distinguish between the different types. Thus, it is possible to optimise the maximum likelihood estimator as well as the accuracy and speed of the computations with a protein length in addition to a unique minInDel pattern. Given that the low-frequency minInDel functions as an active subspace to separate the orthologous regions prior to the high-frequency substitution under selective constraints, it is used to calculate the maximum likelihood estimate of the evolutionary distance (d) with nonzero Findel likelihood acquisition by comparative sequence analysis.

5 $$ d=\widehat{d}+\widehat{\ell}\left({F}_{indel}\right) $$

in which the phylogenetic distance ($\widehat{d}$) was estimated by the maximum likelihood method. Based on a distance matrix generated from pairwise gapless sequence alignments, the minInDel-corrected distance (*d*) was calculated by adding likelihood estimates $\widehat{\ell}$(*F*_indel_) for the pairwise distance between different virus types. The minimum evolution (ME) tree was searched using the Neighbor-Joining (NJ) algorithm [27], and the Close-Neighbor-Interchange algorithm [28]. The tree scaled with branch lengths measuring the number of minInDel and substitutions per site, and was compared to the NJ guide tree with branch lengths measured only in the number of substitutions per site.

### Evaluation of F_indel_ values for inferring the evolutionary distance between genes

To evaluate the effect of minInDel on the viral polyprotein gene sequences, the minInDel frequency in Eq. (1) was modified by multiplying by three times the number of minInDel amino acids (*I*_A_), which corresponds to a multiple of three nucleotides (*I*_N_), but not one or two nucleotides, which would cause reading frameshifts in the nucleotide sequences.

6 $$ {F}_{indel}=\frac{1}{n}\sum \limits_{i=1}^n\sqrt{{\left(\frac{I_N}{I_N+{S}_N}\right)}_i^2} $$

The consensus coding sequence was generated from Geneious 10.2.3 (Biomatters Ltd., Auckland, New Zealand) using a BLOSUM62 matrix, in which the third nucleotide positions of the stop codons (UAA, UAG, and UGA) were replaced with the ambiguous “n” character. The pairwise alignment was performed using Clustal W in the TranslatorX server [29]. After removing InDels (gaps) and ambiguous codons, including the “n” character, the number of non-synonymous substitutions (*S*_N_) was determined by one-half of the sum of the binary numbers (1, non-synonymous substitution; and 0, synonymous substitution) in the aligned codon positions. When these values were substituted into Eq. (1), appropriately modified Eqs. (4, 5) were used to evaluate the genetic properties of the mutations, including minInDel, nonsynonymous substitution rate, and potential biases in inferring the evolutionary distance computed across the coding region.

## Results

### Minimum InDel patterns of the Zika virus and dengue virus

**Fig. 1:**
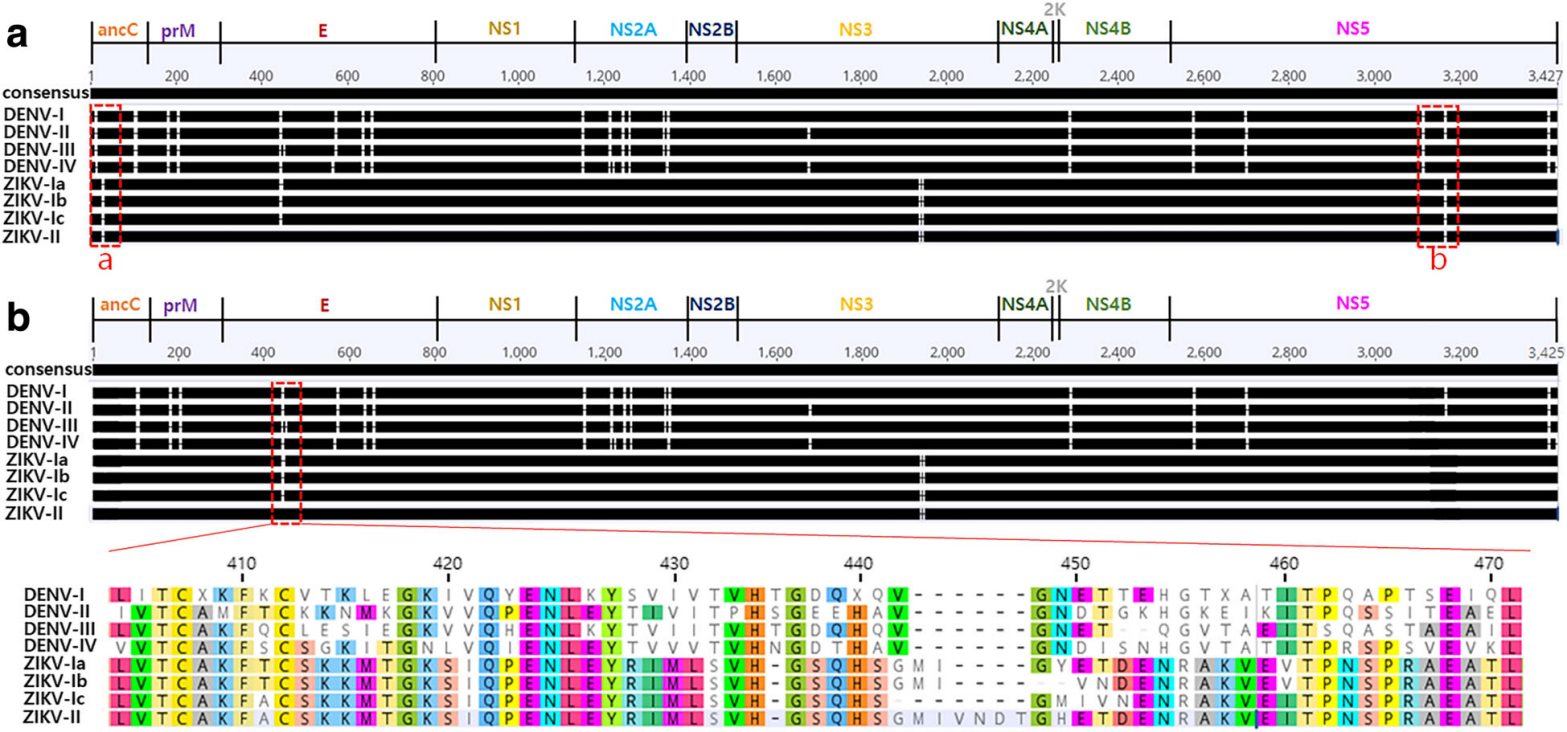
Consensus amino acid sequence alignments and minInDel positions for Zika virus (ZIKV) types and Dengue virus (DENV) types. **a** A conventional method for multiple sequence alignment with gaps resulting in an increase in the length of the consensus sequence to 3427 amino acid residues. **b** A minimum InDel (minInDel) method for reducing the ambiguity of highly weighted gaps in parts a and b (boxed) of the upper panel, thereby resulting in an unweighted consensus sequence that has a maximum length of 3425 amino acid residues for the viral polyproteins. The unique minInDel position in the envelope E protein shared by ZIKV type I and DENV types is enlarged below

Full polyprotein genome sequences of ZIKV strains isolated from 1947 to 2017 were achieved with the records of the isolation site (country), isolation year, host (mosquitos, primates, cell lines, and human), test sample, and clinical information (Additional file 1). The minInDel patterns based on the length of the consensus sequences of the ZIKV and DENV strains divided the ZIKV strains into two types (ZIKV-I and -II), according to the variation in the length of the E protein (Fig. 1). The two ZIKV types were found to co-circulate in the Zika forest of Uganda in 1947. They were clearly distinguishable from the minInDel patterns of the DENV strains, which are divided into four types (DENV-I to -IV). The results show the unique minInDel patterns in the NS3 and NS5 proteins, which is consistent with the current four types of DENV [30]. The minInDel maps based on an unweighted consensus (3425 a.a.) removed ambiguous InDel variables from a weighted MSA, thus increasing the sequence length (3427 a.a.) in parts a and b (boxed) of the upper panel of Fig. 1a. This result makes it clear that the four types of DENV and ZIKV-I have a shared minInDel character in a hypervariable region of the E protein (Fig. 1b). This finding shows that ZIKV and DENV partially inherit the hierarchic structure of the E protein, though the gapless MSA fails to predict key substitutions.

### Spearman correlation coefficients of minInDel patterns

Spearman correlation coefficients for the ranks of normalised data of the protein length in the collected datasets of ZIKV and DENV types were analysed in comparison with the consensus (Table 1). The probability of a monotonic relationship between the average ranks given by the consensus and minInDels was measured with the *P*-value. The low probability for distinguishing between the consensus and sample size accounts for a low number of InDel variables, which are rank-ordered in the polyprotein. The Spearman correlation tests yielded the same results for DENV-I and DENV-III (ρ = 0.5455, *P* = 0.0827) and for DENV-II and DENV-IV (ρ = 0.5227, *P* = 0.0990), which are distinct from each other with respect to the minInDel values in the NS3 and NS5 proteins. Comparing the results shows that the length of the normalisation procedure increases the statistical power of the rank analysis with a single amino acid minInDel fixed in the small NS3 protein rather than the distribution of multiple random variables in the large NS5 protein. In other words, a valid minInDel value gives the higher rank to the Spearman correlation method, regardless of the protein length. It is, however, difficult to know how and where to start, due to the lack of reports of the co-circulation of the four DENV types at any time and any place [30].

**Table 1:** Spearman correlation coefficients for the minInDel patterns of DENV and ZIKV types

minInDel pattern	Prototype	Rho (ρ)*	*P* value	Simpson’s index of Diversity
DENV-I	DENV/Japan/1943/AB074760	0.5455	0.0827	
DENV-II	DENV/PapuaNewGuinea/1944/KM204118	0.5227	0.0990	
DENV-III	DENV/Philippines/1956/KU050695	0.5455	0.0827	
DENV-IV	DENV/Philippines/1956/KR011349	0.5227	0.0990	
ZIKV-I	ZIKV/Uganda/1947/AY632535	0.7727	0.0053	0.0988
ZIKV-II	ZIKV/Uganda/1947/KX377335	0.8750	0.0004	

*Rho was calculated by Spearman correlation with the consensus sequence of all types

In contrast, the ZIKV strains isolated from the Zika forest of Uganda in 1947 and from China in 2016 had four distinct minInDel patterns in the E proteins that were homologous to that of DENV. The effect of the minInDel length scale on characterisation of the ZIKV types was evaluated by varying the observed E protein length from 504 to 497 amino acid residues. The Spearman correlation results of each of the full polyproteins compared to the consensus sequence of DENV and ZIKV proved that a single or multiple amino acid loss from the 504-amino acid E protein of ZIKV-II (ρ = 0.8750 and *P* = 0.0004) displays a strong relationship with the generation of various subtypes of ZIKV-I (ρ = 0.7727 and *P* = 0.0053 for an E protein less than 503 a.a.), including the ZIKV subtypes, Ia (500 a.a.), Ib (498 a.a.), and Ic (497 a.a.). The subtypes Ia and Ib co-circulated in the Zika forest of Uganda in 1947, and the new subtype Ic was isolated from China in 2016. The probability was calculated to have a strong monotonic pattern only with the consensus (504 a.a.), but increased with a decrease in the E protein length. This finding implicated that the various subtypes of ZIKV could derive from insertion and deletion of single or multiple amino acids in the E protein. However, the measured Simpson’s index of diversity was low (0.099) for the two types of ZIKV-I and -II. The reason is that the reported ZIKV cases have reflected more attention paid to Asian/American strains than African strains.

It was previously suggested that the ancestral ZIKV strain that originated from Africa has spread to Asia, the Pacific Islands and now to the Americas and beyond; however, this history has not been reflected in studying the patterns of mutation accumulation in the viral gene [23, 24]. The investigators did not encompass the entire range of each ZIKV type at spatial and temporal scales because of the very low capacities for the detection of emergent conditions in most of the African continent [23, 31–33]. Before the twenty-first century, ZIKV infections have been sporadic because false-negative results might happen due to the clinical similarity and cross-reactive response of ZIKV with DENV [10]. The prediction of diversity would be further complicated by viral population changes due to epidemic Asian/American strains and the risk to human health, in which repeated bottleneck effects, genetic drift, and sampling errors can be evoked in their interactions with different host environments. Otherwise, sequencing errors could confound the phylogenetic analyses among the large collection of ZIKV [14]. To reduce any bias that might arise from simple random mutations and sequencing errors, the minInDel pattern analysis on the consensus sequence was useful in finding a unique minInDel pattern in a divergent population by the robustness to non-additive noise at the expense of losing the parametric length. The resulting two main types of ZIKV-I and -II now encompass all of the different ZIKV strains.

### Estimating the minInDel frequency and evolutionary distance corrects the phylogenetic tree of ZIKV

**Fig. 2:**
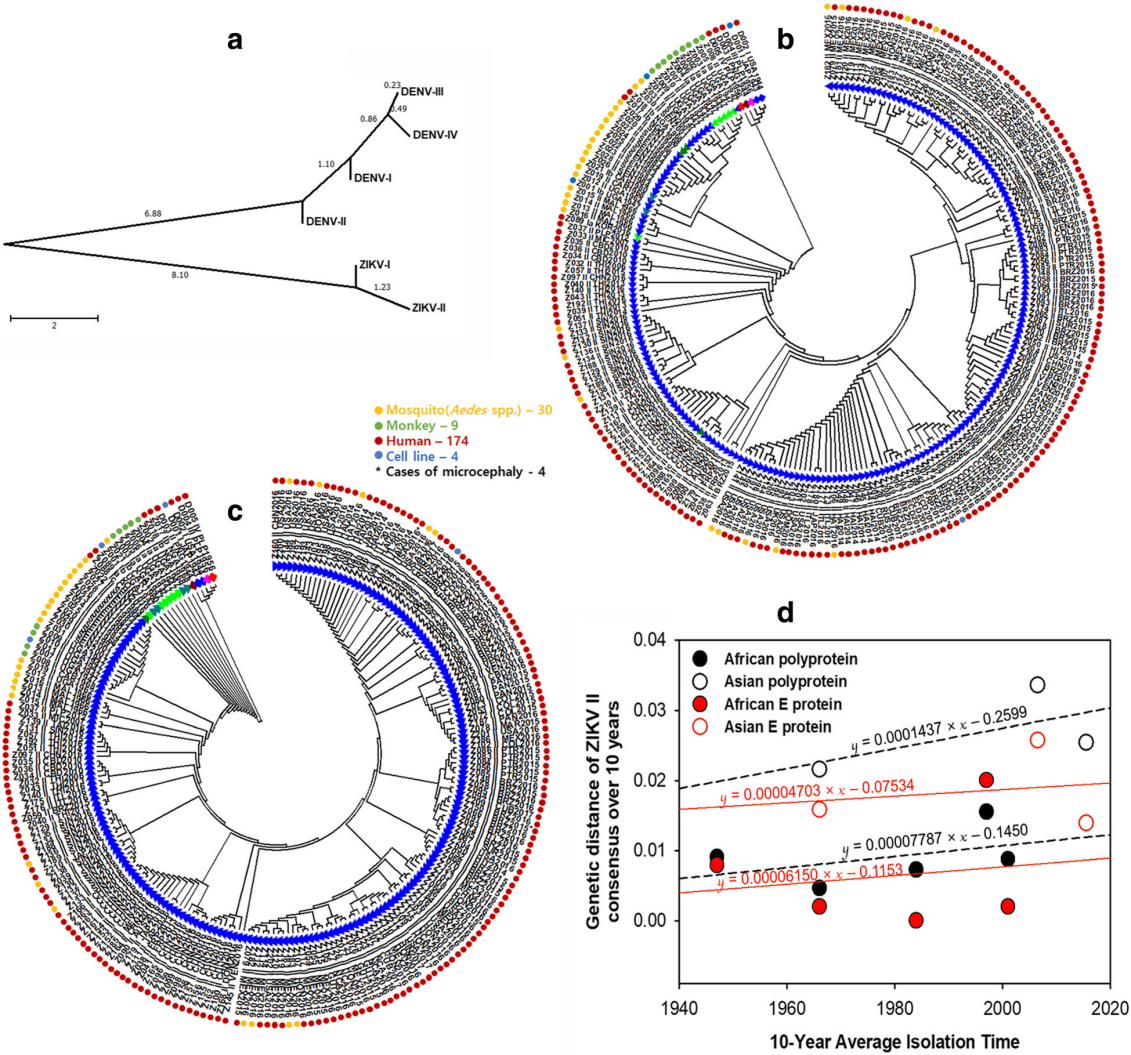
Evolutionary relationships for the polyproteins of the ZIKV and DENV types. **a** A neighbor-joining tree likelihood of distance between the minInDel patterns of ZIKV and DENV types. **b** A topology of the maximum likelihood tree based on a gapless multiple sequence alignment of the full polyprotein sequences of the ZIKV and DENV strains. **c** A topology of a minimum evolution tree with a distance correction by calculating the minInDel frequencies in the polyprotein sequences. Virus types and hosts are differentiated by different colours of triangles and circles shown at the front and end of each strain code, and four cases of microcephaly are indicated by asterisks. The neighbor-joining trees with branch lengths are shown in Additional file 3. **d** The 10-year average genetic distance plots of the African and Asian strains that belong to ZIKV type II

In the present study, the minInDel patterns of ZIKV and DENV support the uniqueness of each type in terms of the protein length with respect to the polyprotein organisation. It is simple to measure the minInDel frequency, distribution probability, and evolutionary distance as low as an amino acid length difference in the polyprotein sequence by the proposed equations. The likelihood estimation of the distance between the minInDel patterns was used to assess the evolutionary relationships among the virus types (Fig. 2a). Except for the minInDel values of ZIKV-I and -II, random amino acid substitutions occurred throughout the polyprotein. The NJ trees obtained from different models for amino acid substitutions only (without InDel) did not distinguish well between ZIKV-I and -II types in some strains of the African lineage: a clade of the Uganda ZIKV-Ia 1947 (KU963573) and ZIKV-II 1947 (LC002520), a clade of the Uganda ZIKV-Ib 1947 (DQ859059) and ZIKV-II 1962 (KY288905), and a clade of the Central Africa Republic ZIKV-Ib 1980 (KF268949) and ZIKV-II 1968 (KF383115) (Fig. 2b). The recent Asian ZIKV-I strains from Korea and China in 2016, which are classified to the subtypes Ia (KY553111) and Ic (MG674719) respectively, are clustered in the Asian lineage of ZIKV-II. Nevertheless, the gapless mode markedly increases the genetic distance of ZIKV among the Asian strains, and unlikely that of African strains, as shown in previous studies [5, 24, 34]. In the history of ZIKV appearing to transcend time and types, Faye and coworkers argued that the acquisition of minInDels that involved an *N*-glycosylation site in the asparagine 154 residue of the E protein is a recurrent event that is important for the infectivity and assembly of flaviviruses [12]. To infer the minInDel bias towards amino acid changes, we introduced the minInDel frequency to correct the phylogenetic tree (Fig. 2c). The branch lengths of the phylogenetic trees inferred by the ME method are shown in Additional file 3. The resulting tree clearly distinguished the ZIKV types which varied with time and space. This result indicates that the unweighted minInDel length in the E protein of ZIKV is the shortest path to calculate the degree, closeness, and betweenness centrality for all of the nodes in a phylogenetic tree. The minInDel distance correction also supported evidence for the presence of a long branch that leads to the new African lineage of ZIKV-II in Senegal between 1997 and 2001, which is consistent with results of previous studies that suggested that recombination has possibly occurred between different genotypes in Africa [8, 10, 12, 16, 35].

A minInDel-corrected tree topology showed that distinct ZIKV (sub)types of African strains have evolved steadily over time before the year 2000. Except for the minInDel value, amino acid substitution rates of ZIKV-II strains significantly increased after its spread to Asian Pacific American countries. This incident was first detected from the Malaysian strains in 1966, but it was difficult to track the original source and global expansion of the Asian strains because of the lack of genome information and knowledge on the distribution and diversity of the various ZIKV types which greatly vary among the lineages by the host ranges and geographical area. The substitution rate of the Asian ZIKV-II strains highly increased from the Malaysia strains in 1966 and gradually decreased during epidemics in the past 10 years. Based on a molecular clock, a linear evolution model assumes that mutations accumulate one after the other, but it is not valid for genetic drift, which causes shifts in alleles and phenotypes. The biased substitution rate of ZIKV-II in the Asian lineage could be due to the spread of a new strain with a high proportion of slightly deleterious mutations, which contribute to adaptive molecular evolution in the vector host environments of the virus, as a general mechanism of evolution [23, 34, 36–38]. The Asian lineage of ZIKV-II has been therefore divided into two sublineages from different hosts, i.e., mosquitos and humans, as mentioned previously [39]. The observed evolutionary distance between the two Asian sublineages of ZIKV-II is large, but it is not as high as between the Asian and African lineages. Except for the new African II lineage strain (KF383118) which has undergone recombination with distantly related strains, the evolutionary rates of the Asian and African ZIKV-II lineages were independently estimated from the 10-year average genetic distance plots of 200 strains, as shown in Fig. 2d. When the calculated genetic distance data were extrapolated, the time of their origin was estimated at approximately 270 years ago based on the full polyprotein sequence, in which mutation accumulation in the E protein was relatively slow in both lineages. This inference is approximately 100 ~ 200 years older than those estimated based on a rate of ~ 10^− 3^ substitutions per site per year in the full genome sequences of the Asian strains [5, 23, 24], and 0.212 substitutions per site per year in the E gene sequences of the two African ZIKV-II strains, which were generated by recombination [11]. These results indicate the genetic variation in ZIKV-II among the different lineages in the assessed regions.

The minInDel frequency per mutation between ZIKV-I and -II (*f*_indel_ = 0.0191) was invariable, which leads to a monotonic pattern. This value was approximately 3 times higher than the average minInDel frequency (0.0059) among the four DENV types, within a range of 0.0019 to 0.0101. The minInDel frequencies between the ZIKV and DENV types were also high, which caused the maximum likelihood estimation of the distance between the different virus species to vary largely. Most likely, the minInDel frequency of the viral polyprotein continuously varies within a species, but not between different species. Although the minInDel patterns of the full polyproteins are highly diverse between ZIKV and DENV, the minInDel pattern of the E protein is relatively conserved through evolution.

**Fig. 3:**
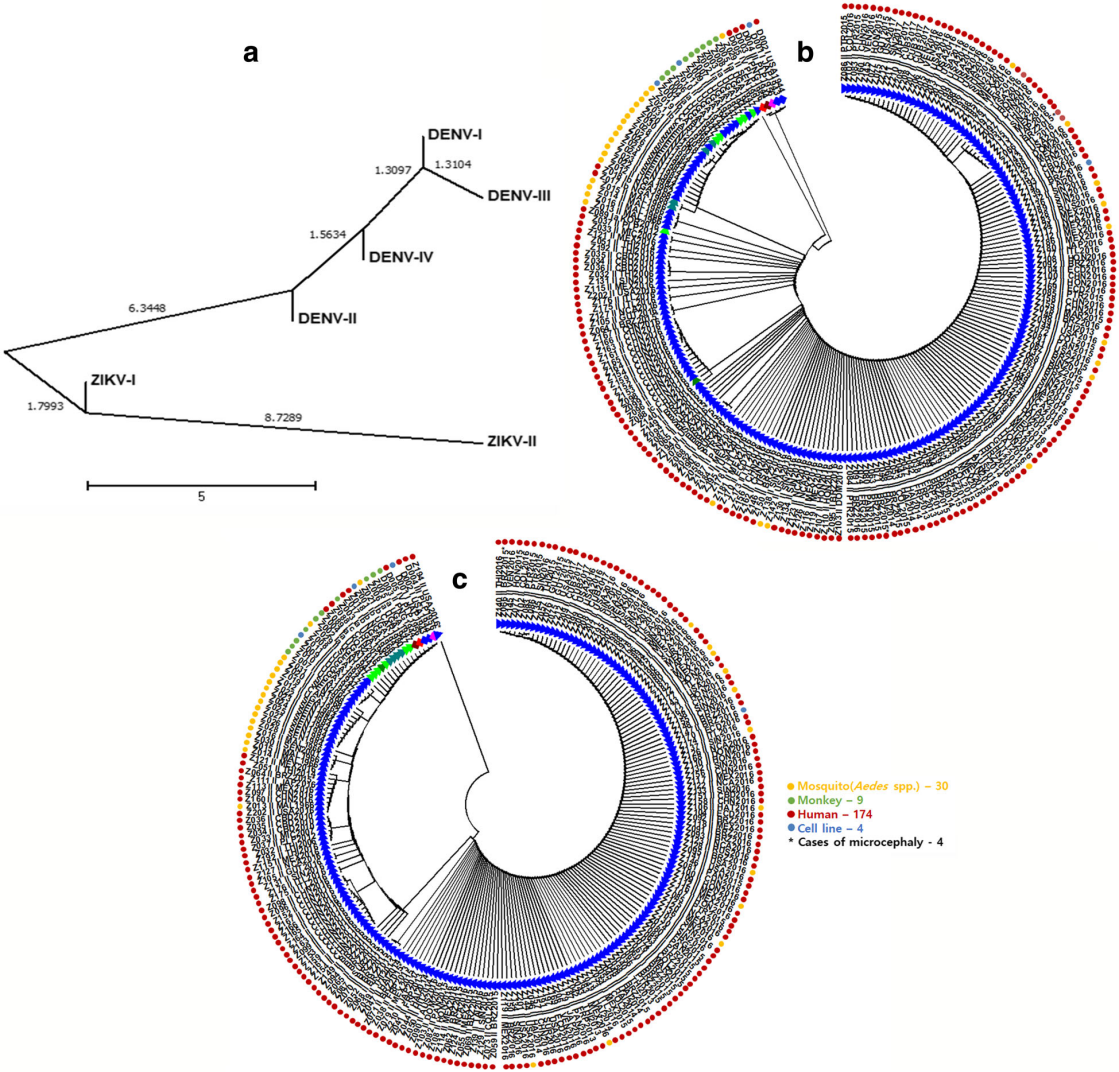
Evolutionary relationships for the E proteins of the ZIKV and DENV types. **a** A neighbor-joining tree likelihood of distance between the minInDel patterns in the E proteins of ZIKV and DENV types. **b** A topology of the maximum likelihood tree based on a gapless multiple sequence alignment of the E protein sequences of the ZIKV and DENV strains. **c** A topology of a minimum evolution tree with a distance correction by calculating the minInDel frequencies in the E protein sequences. Virus types and hosts are differentiated by different colours of triangles and circles shown at the front and end of each strain code, and four cases of microcephaly are indicated by asterisks

In previous studies, the hypervariable region in the E protein (which encompasses the unique minInDel positions) was used for detection and typing of ZIKV and DENV [12, 40]. Its minInDel distribution probability along the polyprotein sequence is low, but the minInDel difference in the E protein is important to distinguish between ZIKV-I and -II in the evolutionary relationship with the DENV types (Fig. 3a). When pairwise deletion of gaps in sequence comparisons was selected, the ME tree topology showed that a recombinant E allele in the genome of the Senegal/1997 strain (KF383117) is connected to species of DENV and ZIKV without distinguishing between different types (Fig. 3b). Using this tree as a guide tree, a minInDel-corrected ME tree was created to show that the E protein has a unique minInDel pattern for various types of ZIKV and DENV, which derived from a common ancestor and continue to differentiate between different mutations during epidemic spread of ZIKV-II, particularly in Brazil and USA between 2015 and 2016 (Fig. 3c). In fact, ZIKV and DENV have a similar history, epidemiology, transmission, and clinical presentation [41, 42]. However, it is still difficult to assess the origins and evolution of the virus types, because the majority of past studies have focused on the Asian/American type, but not the other types in the climate-susceptible areas that could hold potential for selection by the vector host interactions and environments.

### Effects of minInDels on gene sequences

**Fig. 4:**
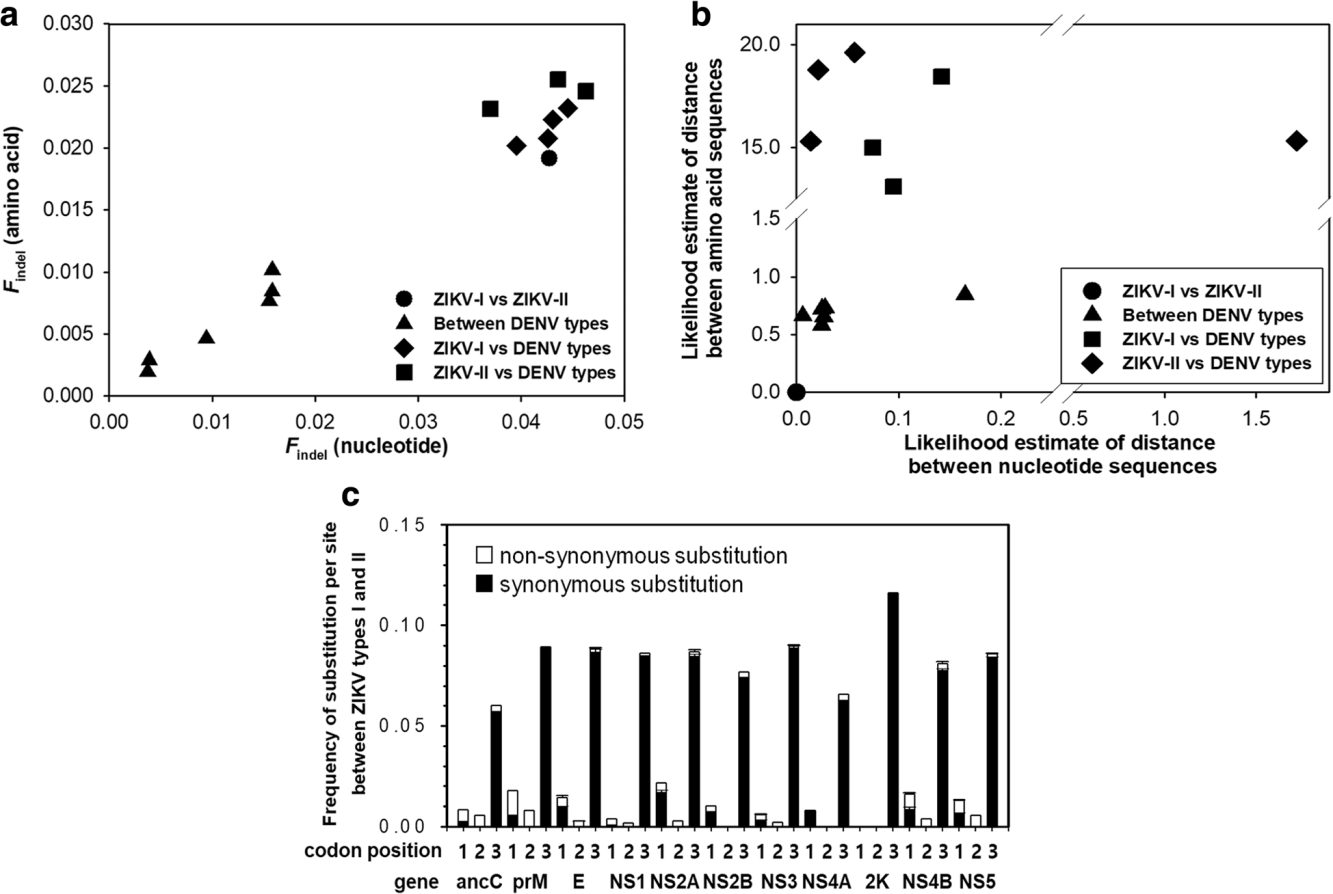
Effects of minInDels on the likelihood estimation of the evolutionary distance for the ZIKV and DENV types. **a** A plot of the minInDel frequencies that result from comparisons of the amino acid sequences and nucleotide sequences of the viral polyproteins. **b** A plot of likelihood estimates that result from comparisons of amino acid sequences and nucleotide sequences of the viral polyproteins. **c** Frequencies of non-synonymous and synonymous substitutions at the codon nucleotide positions of each coding gene of the two ZIKV types

The effects of minInDels on the gene sequence of ZIKV were evaluated by substituting the numbers of minInDel (*I*_A_) and substituted (*S*_A_) amino acids in Eq. (1) with the numbers of minInDel nucleotides (*I*_N_) and nonsynonymous substitutions (*S*_N_) in Eq. (6). The *I*_N_ value is the absolute number of minInDel nucleotides as calculated by multiplying by three the number of minInDel amino acids, whereas the *S*_N_ value is the coding variants that introduce amino acid changes in a protein sequence. Thus, the outcome of Eq. (6) will likely vary by the *S*_N_ values in the consensus gene sequences. Most comparisons of nucleotide sequences resulted in higher minInDel frequencies (*F*_indel_), which significantly reduced the likelihood estimates of the minInDel-corrected distance between the ZIKV and DENV types compared with those of the amino acid sequences (Fig. 4a and b). The minInDel frequencies were largely varied by comparisons of amino acid and nucleotide sequences. Notably, the nucleotide sequence comparison of the two ZIKV types with a low rate of non-synonymous substitution yielded a high *F*_indel_ value, which resulted in a marginal effect for the correction of the evolutionary distance estimated by the maximum likelihood method (Additional file 4). Nevertheless, their evolutionary relationships with the DENV types of nucleotide sequences were not apparent at the species levels. The reason is that the similarity between the protein-coding sequences degrades more rapidly at the nucleotide level than at the amino acid level, and thus, alignments for phylogenetic inference and reconstruction of consensus sequences usually provide more accurate information on the amino acid sequence rather than the nucleotide sequence [29]. This finding supports that amino acid sequence comparisons have distinct advantages over nucleotide sequence comparisons for the analysis of minInDels and substitutions in the viral polyproteins. The minInDel frequencies and likelihood estimates of distance between amino acid sequences are sufficiently sensitive to distinguish between different types at the species level.

Excluding the minInDel positions, nonsynonymous and synonymous substitutions have similarly occurred in most coding regions of the two ZIKV types (Fig. 4c). The findings are consistent with the results of the previous studies, which suggest that most gene elements in the ZIKV genome are likely under stabilising selection [15, 23]. This circumstance means that most of the accumulated mutations in the ZIKV strains give rise to conservative substitutions and are evolutionarily neutral from the Uganda prototypes in 1947. This tendency can be expressed by changes in the generation time or mutation rate rather than by changes in the effective population size or patterns of natural selection that will mainly alter the nonsynonymous substitution rates [43]. The gapless nucleotide alignments of ZIKV and the related virus genomes provided no genetic markers with regard to the evolution of the ZIKV types, although the different lineages arose from changes in the substitution rates, recombination sites, and secondary RNA structure of the 3′-untranslated regions [15, 24, 35]. Among the lineages, the extent of difference in the nucleotide substitution rates can be accounted for by temporal changes in nucleotide composition or differences between gene and genome phylogenies that operate on a short time scale. In contrast, a unique minInDel pattern, which is validated by Spearman correlation test and likelihood estimation of evolutionary distance between different types, is considered to be a long-term tendency that is relatively independent of the substitution rate change.

## Discussion

We demonstrated that the minInDel pattern in the E protein of ZIKV is a targetable sequence for the rapid detection and typing of closely related viruses. The Spearman correlation test and likelihood estimation between the minInDel patterns were evaluated not only for strain typing of divergent genotypes but also for evolutionary distance correction. This nonparametric approach is similar to multi-locus sequence typing [44], but the minInDel analysis determines the minInDel frequency expressed as an absolute InDel number per mutation that resulted in an amino acid change in a protein or polyprotein.

When a unique minInDel without regard to its length is fixed in an essential protein that is involved in pathogenesis, it is likely to produce different phenotypes under different environmental conditions. In this respect, the unique minInDel found in the E protein, including the surface protein *N*-glycosylation site (Asn154) of ZIKV, provides new insights into the origins and evolution of different types, thus possibly affecting the host infection and spreading of the virion [12]. Without consideration of the minInDel, the amino acid changes are more conservative in the E protein than elsewhere in the polyprotein sequence of ZIKV-II [23]. The origin time of all ZIKV lineages varied from ~ 50 to 180 years ago based on substitution models, such as maximum likelihood and Bayesian inference, without considering the viral protein divergence with the distribution of minInDel [10, 12, 14, 39]. In our dataset for virus types, ZIKV strains share a high sequence identity, with an average of 98.2% between the African ZIKV-I strains, 98.9% between the African ZIKV-II strains, 99.7% between the Asian ZIKV-I strains, and 99.7% between the Asian ZIKV-II strains. The Asian and African lineages of ZIKV-II have 97.1% identity, with a range between 94.6 and 99.9% identities obtained from aligned sequences. In contrast, the total number of pairwise sequence alignments of ZIKV-II strains with ZIKV-I strains exhibit an average identity of 96.9% (range 95.1~ 100%), which is similar between the Asian and African lineages of ZIKV-II. We thought that divergence of the virus lineages would be influenced by the distribution of different virus types, with different mutation rates depending on molecular diversity, geographic distribution and host range of mosquito-specific viruses. Among 212 ZIKV strains which were studied, 11 ZIKV-I strains and a recombinant ZIKV-II strain from Senegal in 2001 were excluded because their numbers were insufficient to estimate genetic distance by the consensus. Based on the consensus polyprotein sequence of ZIKV-II, the divergence time of the African and Asian lineages was estimated at 270 years ago, earlier than previously thought.

Unlikely a phylogenetic tree based on the substitution rate, the minInDel-corrected evolutionary tree topology clearly showed that the branching off of ZIKV-II towards the Asian and African lineages was seemingly independent of the African lineage of ZIKV-I. However, it is still difficult to assess the origin and evolution of the other types and lineages with the unique minInDel pattern in the E protein because there are a limited number of reported ZIKV genomes. There is still much to be learned about the evolution of different type viruses with the unique minInDel character in the E protein, in particular about its dynamics across hosts, species, and different lineages of the virus types, and how selection acts on phenotypic traits created by mutations.

## Conclusions

In this study, we included 212 polyprotein sequences of ZIKV and 5 reference sequences of DENV in order to determine the virus types and correct evolutionary distances between the viruses by the minInDel pattern analysis. Using the consensus sequence of each protein component, the minInDel map was constructed based on MSA of polyprotein sequences and used to evaluate virus types by Spearman correlation tests after normalization of the protein length. We used the minInDel frequency to calculate likelihood estimates of evolutionary distance between the virus types and to correct the maximum likelihood tree. Through this work we presented a new method of the minInDel pattern analysis for determination and validation of the virus types with a unique minInDel character in the E protein. This method can be useful in developing a rapid detection method to improve the global maps in suitable environments for infection and transmission.

## Additional files

Additional file 1: The details for the DENV and ZIKV strains used in this study. (DOCX 33 kb)

Additional file 2: Lengths of consensus amino acid sequences. (DOCX 15 kb)

Additional file 3: Neighbor-joining trees with branch lengths for the full polyprotein sequences of the Zika and Dengue viruses. (a) A maximum likelihood tree with branch lengths that result from a gapless multiple sequence alignment of the full polyproteins of the ZIKV and DENV strains. (b) A minimum evolution tree with branch lengths corrected by the minInDel frequencies in the viral polyprotein sequences. The bars indicate the mutation rate of the InDels or amino acid substitutions per site in the polyprotein. The virus types and hosts are differentiated by the different colours of the triangles and circles shown at the front and end of each strain code, and the four cases of microcephaly are indicated by asterisks. (DOCX 1338 kb)

Additional file 4: Effects of minInDels on the evolutionary relationship between the coding nucleotide sequences of the ZIKV and DENV viruses. (a) A neighbor-joining tree likelihood of the nucleotide sequences of the ZIKV and DENV types analysed using the minInDel frequencies from a binomial distribution of non-synonymous substitutions in pairwise alignments of the viral gene sequences. (b) A maximum likelihood tree with branch lengths that result from a gapless multiple sequence alignment of the complete gene sequences of the ZIKV and DENV strains. (c) A minimum evolution tree with branch lengths corrected by the minInDel frequencies in the viral gene sequences. The bars indicate the mutation rates of the InDels or non-synonymous substitutions per site in the viral gene. Virus types and hosts are differentiated by the different colours of triangles and circles shown at the front and end of each strain code, and the four cases of microcephaly are indicated by asterisks. (DOCX 1473 kb)

## Abbreviations

DENV: Dengue virus
ME: Minimum evolution
minInDel: Minimum insertion and deletion
MSA: Multiple sequence alignment
NJ: Neighbor-joining
ZIKV: Zika virus
