# Supplementary material for: Minimum InDel pattern analysis of the Zika virus
Source: BMC Genomics. 2018 Jul 13;19:535. doi: 10.1186/s12864-018-4935-z (PMC6045892; doi:10.1186/s12864-018-4935-z)
Supplement: Supplementary file 1 — The details for the DENV and ZIKV strains used in this study. (DOCX 33 kb) [file 12864_2018_4935_MOESM1_ESM.docx]

**Additional file 1.** The details for the DENV and ZIKV strains used in this study.

| **Strain** | **Accession no.** | **Country** | **Isolation year** | **Host/Source** |
| --- | --- | --- | --- | --- |
| D001_I_JAP1943 | AB074760.1 | Japan | 1943 | *Cell culture* |
| D002_I_USA1944 | KM204119.1 | USA | 1944 | *Homo sapiens* |
| D003_II_PNG1944 | KM204118.1 | Papua New Guinea | 1944 | *Homo sapiens* |
| D004_III_PLP1956 | KU050695.1 | Philippines | 1956 | *Homo sapiens* |
| D005_IV_PLP1956 | KR011349.2 | Philippines | 1956 | *Homo sapiens* |
| Z001_Ib_UGA1947 | DQ859059.1 | Uganda | 1947 | *Cell culture* |
| Z002_Ia_UGA1947 | KX601169.1 | Uganda | 1947 | *Macaca mulatta* |
| Z003_Ia_UGA1947 | KU955594.1 | Uganda | 1947 | *Macaca mulatta* |
| Z004_Ia_UGA1947 | AY632535.2 | Uganda | 1947 | *Sentinel monkey* |
| Z005_Ia_UGA1947 | KU963573.2 | Uganda | 1947 | *Macaca mulatta* |
| Z006_Ia_UGA1947 | NC012532.1 | Uganda | 1947 | *Sentinel monkey* |
| Z007_II_UGA1947 | KX830960.1 | Uganda | 1947 | *Sentinel rhesus* |
| Z008_II_UGA1947 | LC002520.1 | Uganda | 1947 | *Sentinel monkey* |
| Z009_II_UGA1947 | KX377335.1 | Uganda | 1947 | *Macaca mulatta* |
| Z010_II_UGA1947 | HQ234498.1 | Uganda | 1947 | *Sentinel rhesus* |
| Z011_II_UGA1947 | KU720415.1 | Uganda | 1947 | *Cell culture* |
| Z012_II_UGA1962 | KY288905.1 | Uganda | 1962 | *Aedes africanus* |
| Z013_II_MAL1966 | HQ234499.1 | Malaysia | 1966 | *Aedes aegypti* |
| Z014_II_MAL1966 | KX377336.1 | Malaysia | 1966 | *Aedes aegypti* |
| Z015_II_MAL1966 | KX694533.2 | Malaysia | 1966 | *Aedes sp.* |
| Z016_II_MAL1966 | KX601167.1 | Malaysia | 1966 | *Aedes sp.* |
| Z017_Ib_NGA1968 | HQ234500.1 | Nigeria | 1968 | *Homo sapiens* |
| Z018_Ib_NGA1968 | KU963574.2 | Nigeria | 1968 | *Homo sapiens* |
| Z019_II_CAR1968 | KF383115.1 | Central African Republic | 1968 | *Aedes africanus* |
| Z020_II_SEN1968 | KF383116.1 | Senegal | 1968 | *Aedes luteocephalus* |
| Z021_Ib_CAR1980 | KF268949.1 | Central African Republic | 1980 | *Aedes opok* |
| Z022_II_SEN1984 | KX601166.2 | Senegal | 1984 | *Aedes africanus* |
| Z023_II_SEN1984 | KU955591.1 | Senegal | 1984 | *Aedes africanus* |
| Z024_II_SEN1984 | KU955592.1 | Senegal | 1984 | *Aedes taylori* |
| Z025_II_SEN1984 | KU955595.1 | Senegal | 1984 | *Aedes taylori* |
| Z026_II_SEN1984 | KX198134.2 | Senegal | 1984 | *Aedes africanus* |
| Z027_II_SEN1984 | HQ234501.1 | Senegal | 1984 | *Aedes africanus* |
| Z028_II_SEN1984 | KY348860.1 | Senegal | 1984 | *Aedes africanus* |
| Z029_II_SEN1997 | KF383117.1 | Senegal | 1997 | *Aedes luteocephalus* |
| Z030_II_SEN2001 | KF383118.1 | Senegal | 2001 | *Aedes dalzieli* |
| Z031_II_SEN2001 | KF383119.1 | Senegal | 2001 | *Aedes dalzieli* |
| Z032_II_THI2006 | MG645981.1 | Thailand | 2006 | *Homo sapiens* |
| Z033_II_MIC2007 | EU545988.1 | Micronesia | 2007 | *Homo sapiens* |
| Z034_II_CBD2010 | MH158236.1 | Cambodia | 2010 | *Homo sapiens* |
| Z035_II_CBD2010 | JN860885.1 | Cambodia | 2010 | *Homo sapiens* |
| Z036_II_CBD2010 | KU955593.1 | Cambodia | 2010 | *Homo sapiens* |
| Z037_II_PLP2012 | KU681082.3 | Philippines | 2012 | *Homo sapiens* |
| Z038_II_FRP2013 | KY766069.1 | French Polynesia | 2013 | *Homo sapiens* |
| Z039_II_THI2013 | KX051560.1 | Thailand | 2013 | *Homo sapiens* |
| Z040_II_THI2013 | KX051561.1 | Thailand | 2013 | *Homo sapiens* |
| Z041_II_FRP2013 | KJ776791.2 | French Polynesia | 2013 | *Homo sapiens* |
| Z042_II_FRP2013 | KX369547.1 | French Polynesia | 2013 | *Homo sapiens* |
| Z043_II_THI2013 | KX694532.2 | Thailand | 2013 | *Homo sapiens* |
| Z044_II_HAI2014 | KY415986.1 | Haiti | 2014 | *Homo sapiens* |
| Z045_II_HAI2014 | KY415987.1 | Haiti | 2014 | *Homo sapiens* |
| Z046_II_HAI2014 | KY415988.1 | Haiti | 2014 | *Homo sapiens* |
| Z047_II_HAI2014 | KY415989.1 | Haiti | 2014 | *Homo sapiens* |
| Z048_II_HAI2014 | KY415990.1 | Haiti | 2014 | *Homo sapiens* |
| Z049_II_HAI2014 | KY415991.1 | Haiti | 2014 | *Homo sapiens* |
| Z050_II_HIT2014 | KU509998.3 | Haiti | 2014 | *Homo sapiens* |
| Z051_II_THI2014 | KU681081.3 | Thailand | 2014 | *Homo sapiens* |
| Z052_II_BRZ2015 | MF352141.1 | Brazil | 2015 | *Homo sapiens* |
| Z053_II_COL2015 | KY989971.1 | Colombia | 2015 | *Homo sapiens* |
| Z054_II_MEX2015 | KY631493.1 | Mexico | 2015 | *Homo sapiens* |
| Z055_II_MEX2015 | KY631494.1 | Mexico | 2015 | *Homo sapiens* |
| Z056_II_PTR2015 | MH158237.1 | Puerto Rico | 2015 | *Homo sapiens* |
| Z057_II_THI2015 | KX051562.1 | Thailand | 2015 | *Homo sapiens* |
| Z058_II_BRZ2015 | KX197205.1 | Brazil | 2015 | *Homo sapiens* |
| Z059_II_BRZ2015 | KU940228.1 | Brazil | 2015 | *Homo sapiens* |
| Z060_II_BRZ2015 | KU365780.1 | Brazil | 2015 | *Homo sapiens* |
| Z061_II_BRZ2015 | KU365777.1 | Brazil | 2015 | *Homo sapiens* |
| Z062_II_BRZ2015 | KU365778.1 | Brazil | 2015 | *Homo sapiens* |
| Z063_II_BRZ2015 | KU365779.1 | Brazil | 2015 | *Homo sapiens* |
| Z064_II_BRZ2015* | KU729217.2 | Brazil | 2015 | *Homo sapiens* |
| Z065_II_BRZ2015 | KU729218.1 | Brazil | 2015 | *Homo sapiens* |
| Z066_II_BRZ2015* | KU497555.1 | Brazil | 2015 | *Homo sapiens* |
| Z067_II_BRZ2015 | KX280026.1 | Brazil | 2015 | *Homo sapiens* |
| Z068_II_BRZ2015 | KX197192.1 | Brazil | 2015 | *Homo sapiens* |
| Z069_II_BRZ2015 | KU707826.1 | Brazil | 2015 | *Homo sapiens* |
| Z070_II_BRZ2015 | KU321639.1 | Brazil | 2015 | *Homo sapiens* |
| Z071_II_BRZ2015* | KU527068.1 | Brazil | 2015 | *Homo sapiens* |
| Z072_II_COL2015 | KX087102.2 | Colombia | 2015 | *Homo sapiens* |
| Z073_II_COL2015 | KU820897.5 | Colombia | 2015 | *Homo sapiens* |
| Z074_II_FRG2015 | KU758877.1 | French Guiana | 2015 | *Homo sapiens* |
| Z075_II_GUT2015 | KU501217.1 | Guatemala | 2015 | *Homo sapiens* |
| Z076_II_GUT2015 | KU501216.1 | Guatemala | 2015 | *Homo sapiens* |
| Z077_II_HON2015 | KX694534.2 | Honduras | 2015 | *Homo sapiens* |
| Z078_II_MAR2015 | KU647676.1 | Martinique | 2015 | *Homo sapiens* |
| Z079_II_MEX2015 | KX247632.1 | Mexico | 2015 | *Homo sapiens* |
| Z080_II_PAN2015 | KX156775.2 | Panama | 2015 | *Homo sapiens* |
| Z081_II_PAN2015 | KX156774.2 | Panama | 2015 | *Homo sapiens* |
| Z082_II_PAN2015 | KX156776.2 | Panama | 2015 | *Homo sapiens* |
| Z083_II_PTR2015 | KX087101.3 | Puerto Rico | 2015 | *Homo sapiens* |
| Z084_II_PTR2015 | KU501215.1 | Puerto Rico | 2015 | *Homo sapiens* |
| Z085_II_PTR2015 | KX377337.1 | Puerto Rico | 2015 | *Homo sapiens* |
| Z086_II_PTR2015 | KX601168.1 | Puerto Rico | 2015 | *Homo sapiens* |
| Z087_II_SUR2015 | KU312312.1 | Suriname | 2015 | *Homo sapiens* |
| Z088_Ic_CHN2016 | MG674719.1 | China | 2016 | *Homo sapiens* |
| Z089_Ia_KOR2016 | KY553111.1 | South Korea | 2016 | *Homo sapiens* |
| Z090_II_AUS2016 | KX806557.3 | Australia | 2016 | *Homo sapiens* |
| Z091_II_BRZ2016 | KY441401.1 | Brazil | 2016 | *Homo sapiens* |
| Z092_II_BRZ2016 | KY441402.1 | Brazil | 2016 | *Homo sapiens* |
| Z093_II_BRZ2016 | KY441403.1 | Brazil | 2016 | *Homo sapiens* |
| Z094_II_BRZ2016 | KY014317.2 | Brazil | 2016 | *Homo sapiens* |
| Z095_II_BRZ2016 | KY014320.2 | Brazil | 2016 | *Homo sapiens* |
| Z096_II_CHN2016 | MG674718.1 | China | 2016 | *Homo sapiens* |
| Z097_II_CHN2016 | KY328290.1 | China | 2016 | *Homo sapiens* |
| Z098_II_CHN2016 | MF036115.1 | China | 2016 | *Homo sapiens* |
| Z099_II_CHN2016 | KY967711.1 | China | 2016 | *Homo sapiens* |
| Z100_II_CHN2016 | MF167360.1 | China | 2016 | *Homo sapiens* |
| Z101_II_CHN2016 | KY927808.1 | China | 2016 | *Homo sapiens* |
| Z102_II_COL2016 | MF574578.1 | Colombia | 2016 | *Homo sapiens* |
| Z103_II_DOM2016 | KY014300.2 | Dominican Republic | 2016 | *Homo sapiens* |
| Z104_II_ECD2016 | MF794971.1 | Ecuador | 2016 | *Homo sapiens* |
| Z105_II_GUT2016 | MF801378.1 | Guatemala | 2016 | *Homo sapiens* |
| Z106_II_HAT2016 | MF384325.1 | Haiti | 2016 | *Aedes sp.* |
| Z107_II_HON2016 | MF801381.1 | Honduras | 2016 | *Homo sapiens* |
| Z108_II_HON2016 | MF801384.1 | Honduras | 2016 | *Homo sapiens* |
| Z109_II_HON2016 | MF801387.1 | Honduras | 2016 | *Homo sapiens* |
| Z110_II_HON2016 | KX906952.1 | Honduras | 2016 | *Homo sapiens* |
| Z111_II_JAP2016 | LC219720.1 | Japan | 2016 | *Homo sapiens* |
| Z112_II_MEX2016 | KY648934.1 | Mexico | 2016 | *Aedes aegypti* |
| Z113_II_MEX2016 | KY120349.2 | Mexico | 2016 | *Homo sapiens* |
| Z114_II_MEX2016 | KY120348.1 | Mexico | 2016 | *Homo sapiens* |
| Z115_II_MEX2016 | MH157202.1 | Mexico | 2016 | *Homo sapiens* |
| Z116_II_MEX2016 | MG595216.1 | Mexico | 2016 | *Homo sapiens* |
| Z117_II_MEX2016 | MF801395.1 | Mexico | 2016 | *Homo sapiens* |
| Z118_II_MEX2016 | MF801402.1 | Mexico | 2016 | *Homo sapiens* |
| Z119_II_MEX2016 | MH157208.1 | Mexico | 2016 | *Homo sapiens* |
| Z120_II_MEX2016 | MH157213.1 | Mexico | 2016 | *Homo sapiens* |
| Z121_II_MEX2016 | MG494697.1 | Mexico | 2016 | *Homo sapiens* |
| Z122_II_NCA2016 | KY765318.1 | Nicaragua | 2016 | *Homo sapiens* |
| Z123_II_NCA2016 | KY765317.1 | Nicaragua | 2016 | *Homo sapiens* |
| Z124_II_NCA2016 | MF434516.1 | Nicaragua | 2016 | *Homo sapiens* |
| Z125_II_NCA2016 | MF434521.1 | Nicaragua | 2016 | *Homo sapiens* |
| Z126_II_NCA2016 | MF434517.1 | Nicaragua | 2016 | *Homo sapiens* |
| Z127_II_NCA2016 | MF434522.1 | Nicaragua | 2016 | *Homo sapiens* |
| Z128_II_RUS2016 | MF664436.1 | Russia | 2016 | *Homo sapiens* |
| Z129_II_SIN2016 | KY241695.1 | Singapore | 2016 | *Homo sapiens* |
| Z130_II_SIN2016 | KY241694.1 | Singapore | 2016 | *Homo sapiens* |
| Z131_II_SIN2016 | KY241766.1 | Singapore | 2016 | *Homo sapiens* |
| Z132_II_SIN2016 | KY241693.1 | Singapore | 2016 | *Homo sapiens* |
| Z133_II_SIN2016 | KY241692.1 | Singapore | 2016 | *Homo sapiens* |
| Z134_II_SIN2016 | KY241760.1 | Singapore | 2016 | *Homo sapiens* |
| Z135_II_SIN2016 | KY241675.1 | Singapore | 2016 | *Homo sapiens* |
| Z136_II_SIN2016 | KY241787.1 | Singapore | 2016 | *Aedes sp.* |
| Z137_II_SIN2016 | KY241774.1 | Singapore | 2016 | *Aedes sp.* |
| Z138_II_SIN2016 | KY241779.1 | Singapore | 2016 | *Aedes sp.* |
| Z139_II_TAI2016 | MF692778.1 | Taiwan | 2016 | *Homo sapiens* |
| Z140_II_THI2016 | MH119185.1 | Thailand | 2016 | *Homo sapiens* |
| Z141_II_USA2016 | KY075939.1 | USA | 2016 | *Aedes sp.* |
| Z142_II_USA2016 | KY785468.1 | USA | 2016 | *Aedes sp.* |
| Z143_II_USA2016 | KY075938.1 | USA | 2016 | *Aedes sp.* |
| Z144_II_USA2016 | MF988743.1 | USA | 2016 | *Aedes sp.* |
| Z145_II_VEN2016 | KY693680.1 | Venezuela | 2016 | *Homo sapiens* |
| Z146_II_USA2016 | KX827268.1 | USA | 2016 | *Homo sapiens* |
| Z147_II_BRZ2016 | KX811222.1 | Brazil | 2016 | *Cell culture* |
| Z148_II_BRZ2016 | KY272991.1 | Brazil | 2016 | *Homo sapiens* |
| Z149_II_BRZ2016 | KU926310.1 | Brazil | 2016 | *Homo sapiens* |
| Z150_II_BRZ2016 | KU926309.1 | Brazil | 2016 | *Homo sapiens* |
| Z151_II_CBD2016 | KX247646.1 | Cambodia | 2016 | *Homo sapiens* |
| Z152_II_CHN2016 | KU963796.1 | China | 2016 | *Homo sapiens* |
| Z153_II_CHN2016 | KU866423.2 | China | 2016 | *Homo sapiens* |
| Z154_II_CHN2016 | KX185891.1 | China | 2016 | *Homo sapiens* |
| Z155_II_CHN2016 | KU740184.2 | China | 2016 | *Homo sapiens* |
| Z156_II_CHN2016 | KU761564.1 | China | 2016 | *Homo sapiens* |
| Z157_II_CHN2016 | KU820898.1 | China | 2016 | *Homo sapiens* |
| Z158_II_CHN2016 | KX056898.1 | China | 2016 | *Homo sapiens* |
| Z159_II_CHN2016 | KX266255.1 | China | 2016 | *Homo sapiens* |
| Z160_II_CHN2016 | KU744693.1 | China | 2016 | *Homo sapiens* |
| Z161_II_CHN2016 | KU955589.1 | China | 2016 | *Homo sapiens* |
| Z162_II_CHN2016 | KU955590.1 | China | 2016 | *Homo sapiens* |
| Z163_II_CHN2016 | KX117076.1 | China | 2016 | *Homo sapiens* |
| Z164_II_CHN2016 | KU761561.1 | China | 2016 | *Homo sapiens* |
| Z165_II_CHN2016 | KU761560.1 | China | 2016 | *Homo sapiens* |
| Z166_II_CHN2016 | KU820899.2 | China | 2016 | *Homo sapiens* |
| Z167_II_CHN2016 | KX253996.1 | China | 2016 | *Homo sapiens* |
| Z168_II_DOM2016 | KX766028.1 | Dominican Republic | 2016 | *Homo sapiens* |
| Z169_II_ECD2016 | KX879603.1 | Ecuador | 2016 | *Homo sapiens* |
| Z170_II_ECD2016 | KX879604.1 | Ecuador | 2016 | *Homo sapiens* |
| Z171_II_HON2016 | KX262887.1 | Honduras | 2016 | *Homo sapiens* |
| Z172_II_HON2016 | KY328289.1 | Honduras | 2016 | *Homo sapiens* |
| Z173_II_ITL2016 | KX269878.1 | Italy | 2016 | *Homo sapiens* |
| Z174_II_ITL2016 | KU991811.1 | Italy | 2016 | *Homo sapiens* |
| Z175_II_ITL2016 | KY003153.1 | Italy | 2016 | *Homo sapiens* |
| Z176_II_ITL2016 | KY003154.1 | Italy | 2016 | *Homo sapiens* |
| Z177_II_ITL2016 | KU853012.1 | Italy | 2016 | *Homo sapiens* |
| Z178_II_ITL2016 | KU853013.1 | Italy | 2016 | *Homo sapiens* |
| Z179_II_JAP2016 | LC191864.1 | Japan | 2016 | *Homo sapiens* |
| Z180_II_JAP2016 | LC190723.1 | Japan | 2016 | *Homo sapiens* |
| Z181_II_MEX2016 | KX446950.2 | Mexico | 2016 | *Aedes sp.* |
| Z182_II_MEX2016 | KX446951.2 | Mexico | 2016 | *Aedes sp.* |
| Z183_II_MEX2016 | KX856011.1 | Mexico | 2016 | *Aedes sp.* |
| Z184_II_MEX2016 | KX766029.1 | Mexico | 2016 | *Homo sapiens* |
| Z185_II_MEX2016 | KU922923.1 | Mexico | 2016 | *Homo sapiens* |
| Z186_II_MEX2016 | KU922960.1 | Mexico | 2016 | *Homo sapiens* |
| Z187_II_PAN2016 | KX198135.2 | Panama | 2016 | *Homo sapiens* |
| Z188_II_SIN2016 | KX813683.1 | Singapore | 2016 | *Homo sapiens* |
| Z189_II_SIN2016 | KX827309.1 | Singapore | 2016 | *Homo sapiens* |
| Z190_II_SUR2016 | KY348640.1 | Suriname | 2016 | *Homo sapiens* |
| Z191_II_SUR2016 | KU937936.1 | Suriname | 2016 | *Homo sapiens* |
| Z192_II_THI2016 | KY272987.1 | Thailand | 2016 | *Homo sapiens* |
| Z193_II_UK2016 | KX673530.1 | United Kingdom | 2016 | *Homo sapiens* |
| Z194_II_USA2016* | KU870645.1 | USA | 2016 | *Homo sapiens* |
| Z195_II_USA2016 | KY325464.1 | USA | 2016 | *Homo sapiens* |
| Z196_II_USA2016 | KY325465.1 | USA | 2016 | *Homo sapiens* |
| Z197_II_USA2016 | KY325468.1 | USA | 2016 | *Homo sapiens* |
| Z198_II_USA2016 | KY325469.1 | USA | 2016 | *Homo sapiens* |
| Z199_II_USA2016 | KY325472.1 | USA | 2016 | *Homo sapiens* |
| Z200_II_USA2016 | KY325473.1 | USA | 2016 | *Homo sapiens* |
| Z201_II_USA2016 | KY325476.1 | USA | 2016 | *Homo sapiens* |
| Z202_II_USA2016 | KY325477.1 | USA | 2016 | *Homo sapiens* |
| Z203_II_USA2016 | KY325479.1 | USA | 2016 | *Homo sapiens* |
| Z204_II_USA2016 | KX051563.1 | USA | 2016 | *Homo sapiens* |
| Z205_II_VEN2016 | KX702400.1 | Venezuela | 2016 | *Homo sapiens* |
| Z206_II_VEN2016 | KX893855.1 | Venezuela | 2016 | *Homo sapiens* |
| Z207_II_CUB2017 | MH063262.1 | Cuba | 2017 | *Homo sapiens* |
| Z208_II_CUB2017 | MH063264.1 | Cuba | 2017 | *Homo sapiens* |
| Z209_II_CUB2017 | MF438286.1 | Cuba | 2017 | *Homo sapiens* |
| Z210_II_JAP2017 | LC369584.1 | Japan | 2017 | *Homo sapiens* |
| Z211_II_SIN2017 | MF988734.1 | Singapore | 2017 | *Homo sapiens* |
| Z212_II_USA2017 | MF159531.1 | USA | 2017 | *Homo sapiens* |

*Four strains isolated from patients with microcephaly.
