# Supplementary material for: Minimum InDel pattern analysis of the Zika virus
Source: BMC Genomics. 2018 Jul 13;19:535. doi: 10.1186/s12864-018-4935-z (PMC6045892; doi:10.1186/s12864-018-4935-z)
Supplement: Supplementary file 2 — Lengths of consensus amino acid sequences. (DOCX 15 kb) [file 12864_2018_4935_MOESM2_ESM.docx]

**Additional file 2.** Length of consensus amino acid sequence.

| Protein product | DENV-I | DENV-II | DENV-III | DENV-IV | ZIKV-I | ZIKV-II | Length of consensus |
| --- | --- | --- | --- | --- | --- | --- | --- |
| ancC | 114 | 114 | 114 | 113 | 122 | 122 | 122 |
| prM | 166 | 166 | 166 | 166 | 168 | 168 | 168 |
| E | 495 | 495 | 493 | 495 | 500 | 504 | 504 |
| NS1 | 352 | 352 | 352 | 352 | 352 | 352 | 352 |
| NS2A | 218 | 218 | 218 | 218 | 226 | 226 | 226 |
| NS2B | 130 | 130 | 130 | 130 | 130 | 130 | 130 |
| NS3 | 619 | 618 | 619 | 618 | 617 | 617 | 619 |
| NS4A | 127 | 127 | 127 | 127 | 127 | 127 | 127 |
| 2K | 23 | 23 | 23 | 23 | 23 | 23 | 23 |
| NS4B | 249 | 248 | 248 | 245 | 251 | 251 | 251 |
| NS5 | 899 | 900 | 900 | 900 | 903 | 903 | 903 |
| Polyprotein | 3392 | 3391 | 3390 | 3387 | 3419 | 3423 | 3425 |
