# Supplementary material for: Minimum InDel pattern analysis of the Zika virus
Source: BMC Genomics. 2018 Jul 13;19:535. doi: 10.1186/s12864-018-4935-z (PMC6045892; doi:10.1186/s12864-018-4935-z)
Supplement: Supplementary file 3 — Neighbor-joining trees with branch lengths for the full polyprotein sequences of the Zika and Dengue viruses. (a) A maximum likelihood tree with branch lengths that result from a gapless multiple sequence alignment of the full polyproteins of the ZIKV and DENV strains. (b) A minimum evolution tree with branch lengths corrected by the minInDel frequencies in the viral polyprotein sequences. The bars indicate the mutation rate of the InDels or amino acid substitutions per site in the polyprotein. The virus types and hosts are differentiated by the different colours of the triangles and circles shown at the front and end of each strain code, and the four cases of microcephaly are indicated by asterisks. (DOCX 1338 kb) [file 12864_2018_4935_MOESM3_ESM.docx]

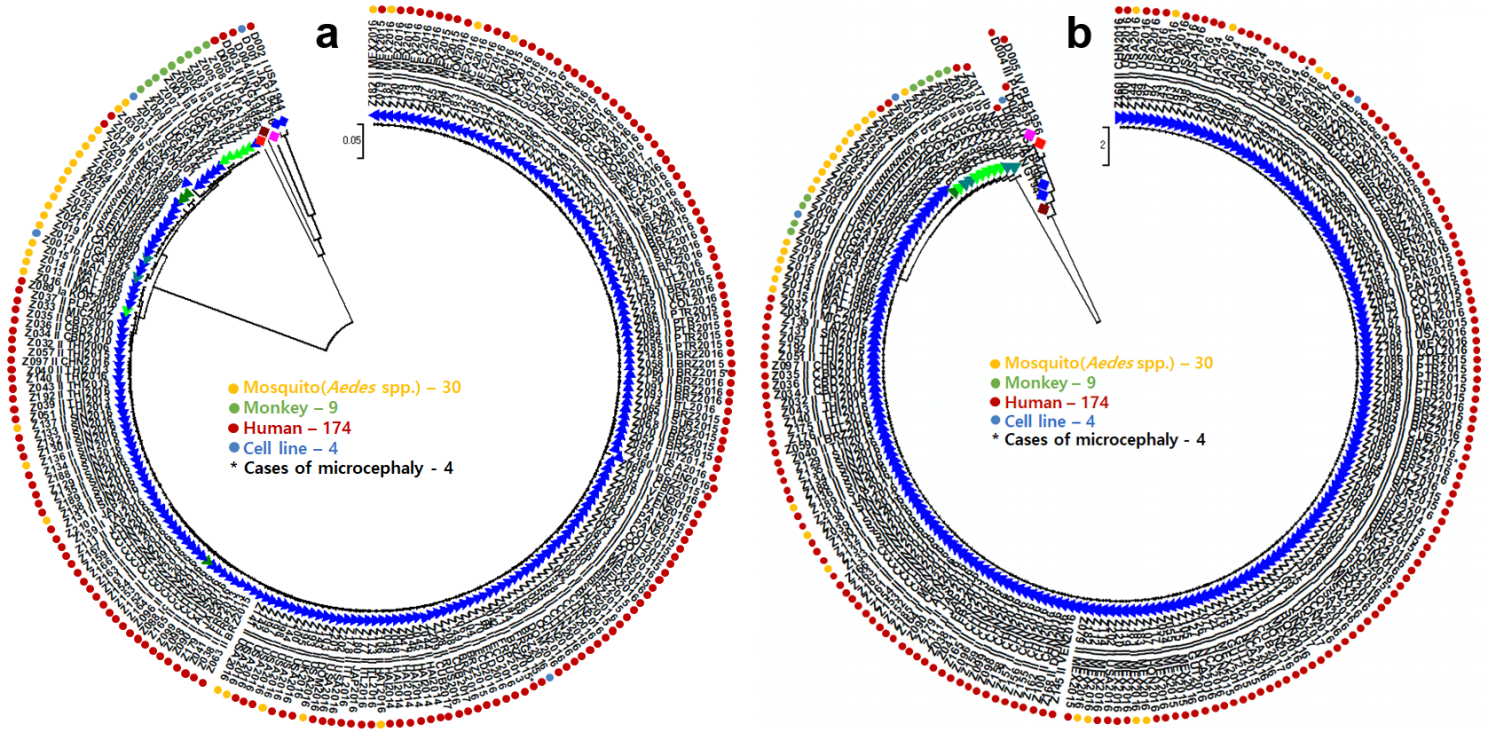


**Additional file 3:** Neighbor-joining trees with branch lengths for the full polyprotein sequences of the Zika and Dengue viruses. (a) A maximum likelihood tree with branch lengths that result from a gapless multiple sequence alignment of the full polyproteins of the ZIKV and DENV strains. (b) A minimum evolution tree with branch lengths corrected by the minInDel frequencies in the viral polyprotein sequences. The bars indicate the mutation rate of the InDels or amino acid substitutions per site in the polyprotein. The virus types and hosts are differentiated by the different colours of the triangles and circles shown at the front and end of each strain code, and the four cases of microcephaly are indicated by asterisks.
