# Supplementary material for: Minimum InDel pattern analysis of the Zika virus
Source: BMC Genomics. 2018 Jul 13;19:535. doi: 10.1186/s12864-018-4935-z (PMC6045892; doi:10.1186/s12864-018-4935-z)
Supplement: Supplementary file 4 — Effects of minInDels on the evolutionary relationship between the coding nucleotide sequences of the ZIKV and DENV viruses. (a) A neighbor-joining tree likelihood of the nucleotide sequences of the ZIKV and DENV types analysed using the minInDel frequencies from a binomial distribution of non-synonymous substitutions in pairwise alignments of the viral gene sequences. (b) A maximum likelihood tree with branch lengths that result from a gapless multiple sequence alignment of the complete gene sequences of the ZIKV and DENV strains. (c) A minimum evolution tree with branch lengths corrected by the minInDel frequencies in the viral gene sequences. The bars indicate the mutation rates of the InDels or non-synonymous substitutions per site in the viral gene. Virus types and hosts are differentiated by the different colours of triangles and circles shown at the front and end of each strain code, and the four cases of microcephaly are indicated by asterisks. (DOCX 1473 kb) [file 12864_2018_4935_MOESM4_ESM.docx]

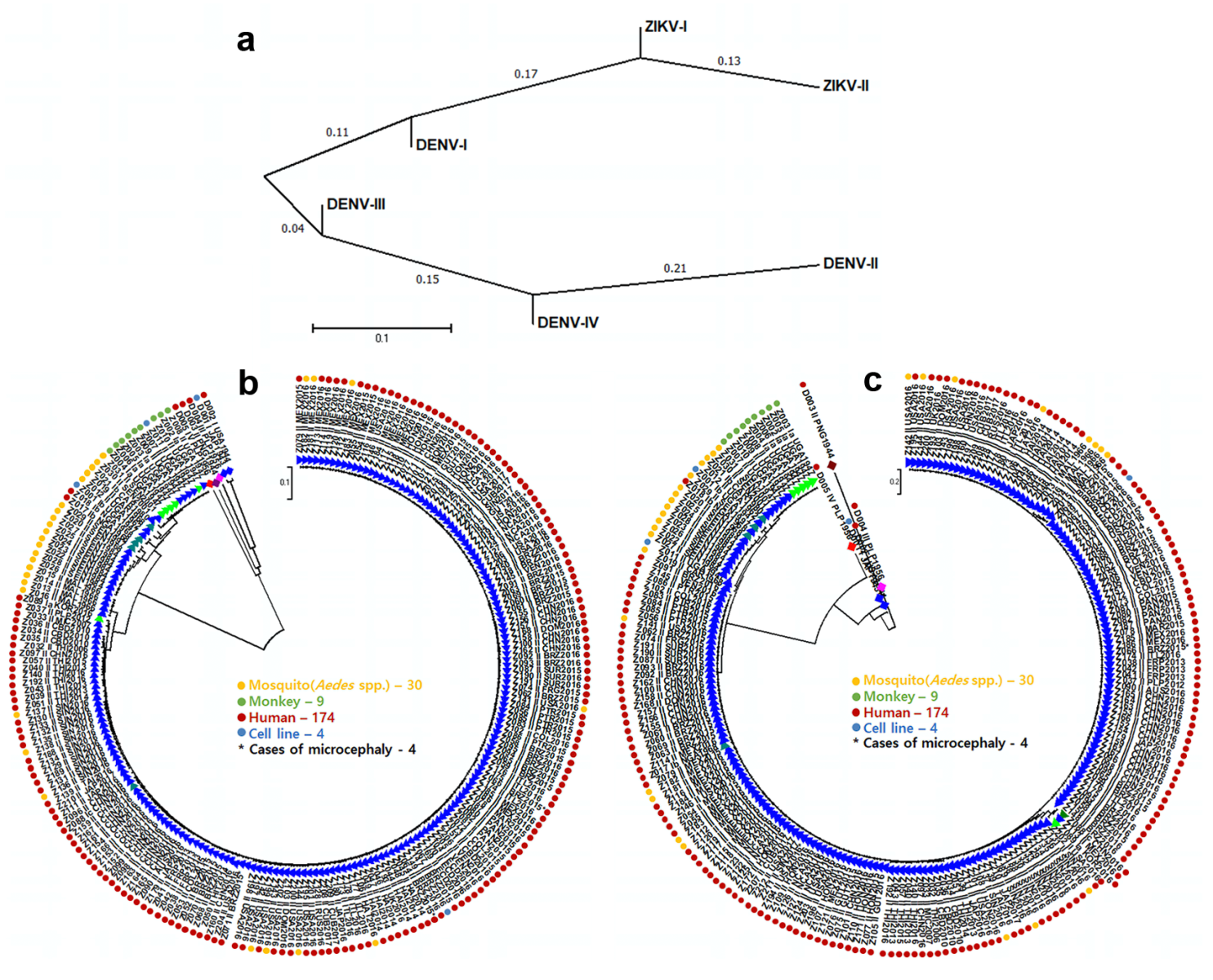


**Additional file 4:** Effects of minInDels on the evolutionary relationship between the coding nucleotide sequences of the ZIKV and DENV viruses. (a) A neighbor-joining tree likelihood of the nucleotide sequences of the ZIKV and DENV types analysed using the minInDel frequencies from a binomial distribution of non-synonymous substitutions in pairwise alignments of the viral gene sequences. (b) A maximum likelihood tree with branch lengths that result from a gapless multiple sequence alignment of the complete gene sequences of the ZIKV and DENV strains. (c) A minimum evolution tree with branch lengths corrected by the minInDel frequencies in the viral gene sequences. The bars indicate the mutation rates of the InDels or non-synonymous substitutions per site in the viral gene. Virus types and hosts are differentiated by the different colours of triangles and circles shown at the front and end of each strain code, and the four cases of microcephaly are indicated by asterisks.
